# Molecular Dynamics Study on Selected Bioactive Phytochemicals as Potential Inhibitors of HIV-1 Subtype C Protease

**DOI:** 10.3390/metabo12111155

**Published:** 2022-11-21

**Authors:** Francis Oluwole Shode, John Omo-osagie Uhomoibhi, Kehinde Ademola Idowu, Saheed Sabiu, Krishna Kuben Govender

**Affiliations:** 1 Department of Biotechnology and Food Science, Faculty of Applied Sciences, Durban University of Technology (DUT), P.O. Box 1334, Durban 4000, South Africa; 2 Department of Chemical Sciences, University of Johannesburg, Doornfontein Campus, P.O. Box 17011, Johannesburg 2028, South Africa; 3 National Institute for Theoretical and Computational Sciences, NITHeCS, Stellenbosch 7602, South Africa

**Keywords:** acquired immunodeficiency syndrome, human immunodeficiency virus, HIV-1 subtype C protease, anti-HIV therapies, molecular dynamics simulation (MDS)

## Abstract

Acquired immunodeficiency syndrome (AIDS), one of the deadliest global diseases, is caused by the Human Immunodeficiency Virus (HIV). To date, there are no known conventional drugs that can cure HIV/AIDS, and this has prompted continuous scientific efforts in the search for novel and potent anti-HIV therapies. In this study, molecular dynamics simulation (MDS) and computational techniques were employed to investigate the inhibitory potential of bioactive compounds from selected South African indigenous plants against HIV-1 subtype C protease (HIVpro). Of the eight compounds (CMG, MA, UA, CA, BA, UAA, OAA and OA) evaluated, only six (CMG (−9.9 kcal/mol), MA (−9.3 kcal/mol), CA (−9.0 kcal/mol), BA (−8.3 kcal/mol), UAA (−8.5 kcal/mol), and OA (−8.6 kcal/mol)) showed favourable activities against HIVpro and binding landscapes like the reference FDA-approved drugs, Lopinavir (LPV) and Darunavir (DRV), with CMG and MA having the highest binding affinities. Using the structural analysis (root-mean-square deviation (RMSD), fluctuation (RMSF), and radius of gyration (RoG) of the bound complexes with HIVpro after 350 ns, structural evidence was observed, indicating that the six compounds are potential lead candidates for inhibiting HIVpro. This finding was further corroborated by the structural analysis of the enzyme–ligand complexe systems, where structural mechanisms of stability, flexibility, and compactness of the study metabolites were established following binding with HIVpro. Furthermore, the ligand interaction plots revealed that the metabolites interacted hydrophobically with the active site amino residues, with identification of other key residues implicated in HIVpro inhibition for drug design. Overall, this is the first computational report on the anti-HIV-1 activities of CMG and MA, with efforts on their in vitro and in vivo evaluations underway. Judging by the binding affinity, the degree of stability, and compactness of the lead metabolites (CMG, MA, CA, BA, OA, and UAA), they could be concomitantly explored with conventional HIVpro inhibitors in enhancing their therapeutic activities against the HIV-1 serotype.

## 1. Introduction

Human immunodeficiency virus (HIV) is one of the most devastating global viral pathogens and a causative agent of acquired immunodeficiency syndrome (AIDS) [1]. HIV defeats the human immune system, making the human defense system susceptible to other opportunistic diseases. According to the World Health Organization, 37.7 million people were living with HIV worldwide in the year 2020 [2]. This figure continues to be staggering because there is currently no permanent cure for this scourge. Nevertheless, recent studies have provided significant knowledge on the action mechanism of HIV, and this has aided the development of drugs to inhibit or control its pathogenic cycle [3]. For instance, the highly active anti-retroviral drugs such as HIV protease inhibitors and integrase inhibitors have aided significant improvement in prognosis outcomes for people living with HIV/AIDS.

HIV protease enzyme (HIVpro) is involved in peptide bond hydrolysis in retroviruses, specifically essential for the life cycle of the virus [4]. Its activity is germane to the replication and eventual release of mature and viable virions [5], and this has made HIVpro a significant target in the development of candidate inhibitors or drugs [6]. The inhibition of this enzyme impedes the viral replication cycle in a manner that results in the release of immature inactive virions [7].

Molecular dynamics simulation (MDS), a computational technique that gives an indication of the nature of interactions and the associated affinity between compatible systems, has been widely used to study interactions between macromolecules (structural proteins) and small molecules such as drugs [8]. To date, drug design remains one of the modern-day applications of MDS to screen, determine, and predict potential therapeutic agents against known druggable targets of diseases [8].

**Figure 1:**
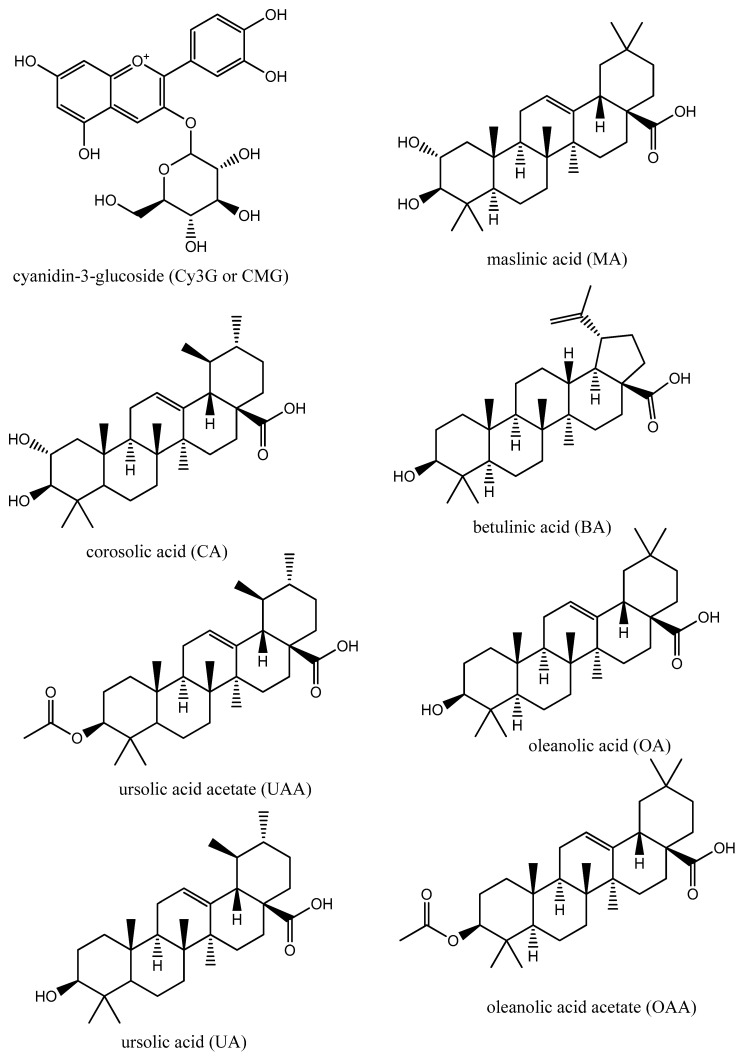
Chemical structures of CMG, MA, CA, BA, UAA, OA, UA, and OAA.

Although many anti-retrovirals (ARVs) have been developed, continuous efforts are needed in sourcing plant-derived, non-synthetic, and easily available inhibitors of druggable targets of HIV such as HIVpro, especially in low-resource countries of the world and, more importantly, due to the prevalence of HIV-1 in Africa. Therefore, considering the foregoing, some selected bioactive nutraceuticals (Figure 1) derived from five underutilised South African-grown medicinal food plants, namely, *Cajanus cajan* [8], *Syzygium aromaticum* [9], *Melaleuca bracteata* ‘Revolution Gold’ [10], *Mimusops caffra* [11], and *Leptospermum petersonii* [12], were computationally explored as inhibitors of HIV-1 protease through MDS. These phytochemicals (cyanidin-3-glucoside (Cy3G), maslinic acid (MA)) possess antiviral activities against viral infections such as HIV, Influenza A and B, and rotavirus replication [13,14,15,16,17,18].

Furthermore, CA has been reported to decrease blood sugar levels, and exhibits antihyperlipidemic, antiviral, and osteoblastic activities [15]. Pavlova et al. reported BA to be active against the herpes simplex virus [16]. Another in vitro study by Tohme et al. demonstrated that UA exhibited antiviral activity against rotavirus, suggesting that UA could be used as a treatment for rotavirus [17]. Jiménez-Arellanes et al., in their study against *Mycobacterium tuberculosis*, reported OA to be effective at displaying a minimum inhibitory concentration (MIC) value of 25 μg/mL against *M. tuberculosis* [19], while UA was reported against *S. mutans* and *S. sobrinus*, with an MIC_50_ of 2.0 μg/mL [20]. These studies emphasised the therapeutic potentials of these triterpenoids. In addition, these metabolites have been reported to have other useful biological properties [21,22,23] such as anticancer, antidiabetes, antiobesity, anti-inflammatory, and antibacterial.

## 2. Methods

### 2.1. HIV-1 Protease and Metabolite Acquisition and Preparation

The X-ray crystal structure of the HIV-1 protease (PDB code: 3U71) was obtained from the RSCB Protein Data Bank [24] and prepared on the UCSF Chimera software package [25], where the monomeric protein was converted to a dimeric structure. The 2D chemical structures of the two FDA-approved drugs, Darunavir (DRV) and Lopinavir (LPV), used as reference standards, as well as the eight metabolites (CMG, MA, CA, BA, UA, UAA, OA, and OAA), were accessed from PubChem [26] and their 3-D structures prepared on the Avogadro software package [27].

### 2.2. Molecular Docking (MD)

The molecular docking software utilised in this study was the Autodock Vina Plugin available on Chimera [28,29], with default parameters. Prior to docking, Gasteiger charges were added to the compounds and the non-polar hydrogen atoms were merged to carbon atoms. The metabolites were then docked into the binding pocket of HIV-1 protease by defining the grid box with a spacing of 1 Å and size of 24 × 22 × 22 pointing in x, y, and z directions. The two FDA-approved drug systems, as well as the eight phytochemicals, were then subjected to molecular dynamics simulations.

### 2.3. Molecular Dynamics Simulation (MDS)

MDS was performed using the graphical processing unit (GPU) version of the AMBER 18 software package, in which the FF18SB variant of the AMBER force field [30] was used to describe the protein. ANTECHAMBER was used to generate atomic partial charges for the ligands (phytocompounds) by utilising the Restrained Electrostatic Potential (RESP) and the General Amber Force Field (GAFF) procedures. The Leap module of AMBER 18 enabled the addition of hydrogen atoms, as well as Na^+^ and Cl^−^ counter ions, for the neutralisation of all systems (the two standard drugs and the eight phytochemicals). The amino acids were numbered as residues 1–198.

The 10 systems were then suspended implicitly within an orthorhombic box of TIP3P water molecules, such that all atoms were within 8 Å of any box edge [29]. An initial minimisation of 2000 steps was carried out with an applied restraint potential of 500 kcal/mol for both solutes (ligand/s and enzyme), and minimisations were performed for 1000 steps using the steepest descent method, followed by 1000 steps of conjugate gradient. An additional full minimisation of 1000 steps was further carried out by the conjugate gradient algorithm without restraint.

A gradual heating MDS from 0 K to 300 K was executed for 50 ps, such that the systems maintained a fixed number of atoms and volume. The solutes within the systems were imposed with a potential harmonic restraint of 10 kcal/mol and collision frequency of 1.0 ps. After heating, an equilibration (500 ps for each system) was conducted; the operating temperature was kept constant at 300 K. Additional features such as pressure were also kept constant, mimicking an isobaric–isothermal ensemble (NPT). The system’s pressure was maintained at 1 bar using the Berendsen Barostat.

The total time for the MDS conducted was 350 ns. In each simulation, the SHAKE algorithm was employed to constrict the bonds of hydrogen atoms. The step size of each simulation was 2 fs and an SPFP precision model was used [30].

### 2.4. Post-Dynamic Analysis

The coordinates of the 10 systems were then saved and the trajectories analysed every 1 ps using PTRAJ, followed by analysis of root-mean-square deviation (RMSD), root-mean-square fluctuation (RMSF), surface area solvent accessibility (SASA), dynamic correlation, and radius of gyration (ROG) using the CPPTRAJ module employed in the AMBER 18 suite.

### 2.5. Binding Free Energy Calculations and Data Analysis

To estimate and compare the binding affinity of the systems, the binding free energy was calculated using the Molecular Mechanics/Generalised Born Surface Area method (MM/GBSA) [31]. The binding free energy was averaged over 100,000 snapshots extracted from the 350 ns trajectory. The binding free energy (ΔG) for each molecular species computed by this method (complex, ligand, and receptor) is represented as follows:

(1) $$\Delta G_{\mathrm{bind}}=G_{\mathrm{complex}}-G_{\mathrm{receptor}}-G_{\mathrm{ligand}}$$

(2) $$\Delta G_{\mathrm{bind}}=E_{\mathrm{gas}}+G_{\mathrm{sol}}-\mathrm{TS}$$

(3) $$E_{\mathrm{gas}}=E_{\mathrm{int}}+E_{\mathrm{vdw}}+E_{\mathrm{ele}}$$

(4) $$G_{\mathrm{sol}}=G_{\mathrm{GB}}+G_{\mathrm{SA}}$$

(5) $$G_{\mathrm{SA}}=\gamma \mathrm{SASA}$$

The term E_gas_ (Equation (3)) denotes the gas-phase energy, which consists of the internal energy E_int_, coulombic energy E_ele_, and the van der Waals energies E_vdw_. The E_gas_ was directly estimated from the FF14SB force field terms. Solvation free energy, G_sol_ (Equation (4)), was estimated from the energy contribution from the polar states, G_GB_, and non-polar states, G_SA_. The non-polar solvation energy, G_SA_, was determined from the solvent accessible surface area (SASA), using a water probe radius of 1.4 Å, whereas the polar solvation, G_GB_, contribution was estimated by solving the $G_{\mathrm{bind}}$ equation. S and T denote the total entropy and temperature of the solute, respectively.

All raw data plots were generated using the Origin data analysis software [32].

## 3. Results and Discussion

The docking scores showed the fitness of the ligands into the active site of the enzyme and the more negative the value, the better the fitness of the ligands [33]. As shown in Table 1, the docking scores for the compounds ranged from −8.1 kcal/mol to −9.9 kcal/mol, with five of the compounds (CMG, MA, CA, OA, UAA) having better scores and binding affinity for the enzyme than the two FDA-approved drugs.

As molecular docking only measures the geometric fitness of ligands at the active site of a protein, the metabolites were further subjected to MDS over a period of 350 ns to assess the binding free energy of each system. The more negative the values of binding free energy, the better the binding affinity and interactions between the enzyme and the ligands [34]. In drug design, binding free energy not only accurately predicts how strongly a potential drug or whether a compound will bind to a protein target, but also measures the binding affinity between the receptor (enzyme) and the ligand [35]. The binding free energies of LPV and DRV and the study metabolites are presented in Table 2. Binding energies of −44.571 and −40.4943 kcal/mol were observed for LPV and DRV, respectively, relative to between −40.165 to −57.890 kcal/mol obtained for the study compounds, with the highest affinities observed with CMG (−57.890 kcal/mol), followed by MA (−48.134 kcal/mol).

### 3.1. Stability, Compactness, and Flexibility of HIV-1pro Apo and HIV-1pro Bound Systems

**Figure 2:**
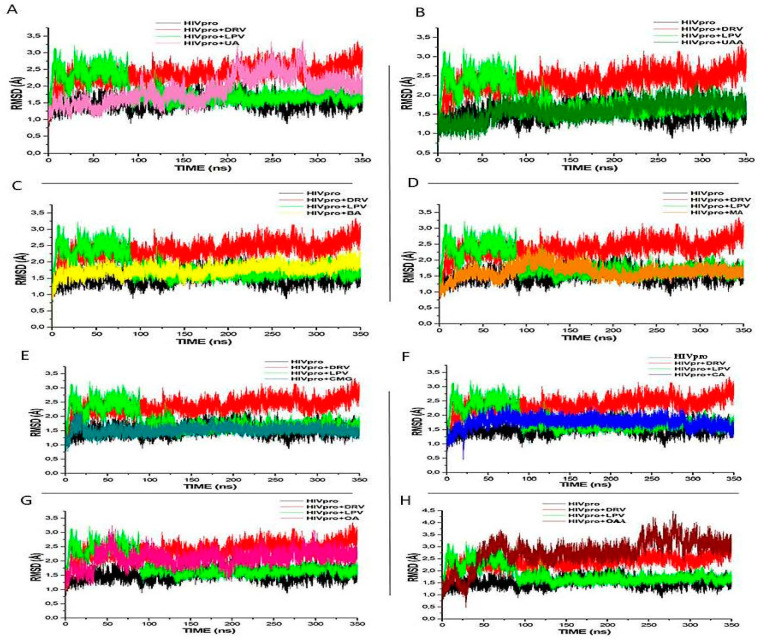
Comparative RMSD profiles of alpha-C atoms of the HIVpro with LPV, DRV, and (**A**) UA, (**B**) UAA, (**C**) BA, (**D**) MA, (**E**) CMG, (**F**) CA, (**G**) OA, and (**H**) OAA systems over a 350 ns molecular dynamics simulation.

To understand the structural stability of a protein complex and the reliability of the MDS, the RMSD, RMSF, and RoG of the backbone atoms of the study compounds’ complexes with HIV-1pro were evaluated. The RMSD gives an indication of the protein stability upon ligand binding, with lower RMSD values indicative of more or better stability of the protein–ligand complex [34,36,37]. In this study, the average RMSD values are within the acceptable limit of <3 Å (Figure 2), thereby supporting the proficiency and reliability of the MDS executed over the 350 ns evaluation period. More specifically, the average RMSD values for the C-alpha atoms of the structures were HIVpro (1.349 Å), DRV (2.235 Å), LPV (1.772 Å), CA (1.632 Å), OA (2.124 Å), OAA (2.783 Å), UA (1.673 Å), UAA (1.513 Å), BA (1.503 Å), MA (1.521 Å), and CMG (1.205 Å). Notably, the lowest RMSD values were observed with CMG, CA, MA, and UAA, denoting both greater stability of the resulting complex with HIVpro in each case and stronger binding affinities. Conversely, while DRV had the highest RMSD value, higher than the mean RMSD value (1.993 Å) for the study compounds, LPV showed some degree of stability after 90 ns of MDS. Generally, compared with the HIVpro apo system, the CMG, BA, UAA, and CA had the lowest RMSD values, indicative of their proficient stability at the binding site of the enzyme (Figure 2). The observations regarding RMSD values of the study ligands in this study are in agreement with previous studies that a lower RMSD values depicts a more stable system [37,38].

**Figure 3:**
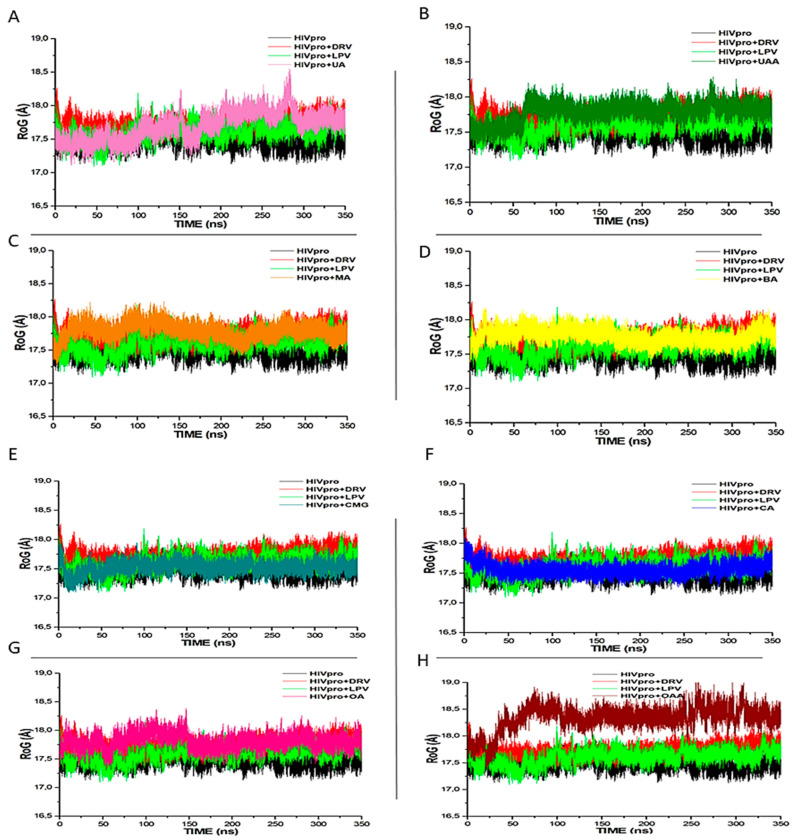
RoG profiles of protein backbone atoms of HIVpro, with LPV, DRV, and (**A**) UA, (**B**) UAA, (**C**) BA, (**D**) MA, (**E**) CMG, (**F**) CA, (**G**) OA, and (**H**) OAA systems over a 350 ns molecular dynamics simulation period.

The RoG is a measure of the structural compactness of a system and is usually employed to study the kinetics, thermodynamics of protein folding, and stability of biomolecular structures [35] following ligand binding with a receptor. The lower the RoG values, the more compacted and stable the receptor–ligand complex [39]. In this study, the mean RoG values for each system are HIVpro (17.362 Å), DRV (17.753 Å), LPV (17.512 Å), CMG (17.478 Å), CA (17.684 Å), OA (17.873 Å), OAA (18.456 Å), UA (17.687 Å), UAA (17.764 Å), MA (17.656 Å), and BA (17.642 Å) (Figure 3). Similar to DRV and LPV complexes, the CA-, MA-, and BA-bound systems were observed to have low RoG values, while the OAA-bound system had the least stability and compactness, as indicated by its RoG, RMSD, and binding free energy in comparison to other ligands. Furthermore, consistent with both the binding free energy and RMSD values, CMG had the lowest RoG value, even lower than DRV and LPV (Figure 3). This study is the first to report the CMG as suggestive of its greater compactness relative to other investigated ligands.

**Figure 4:**
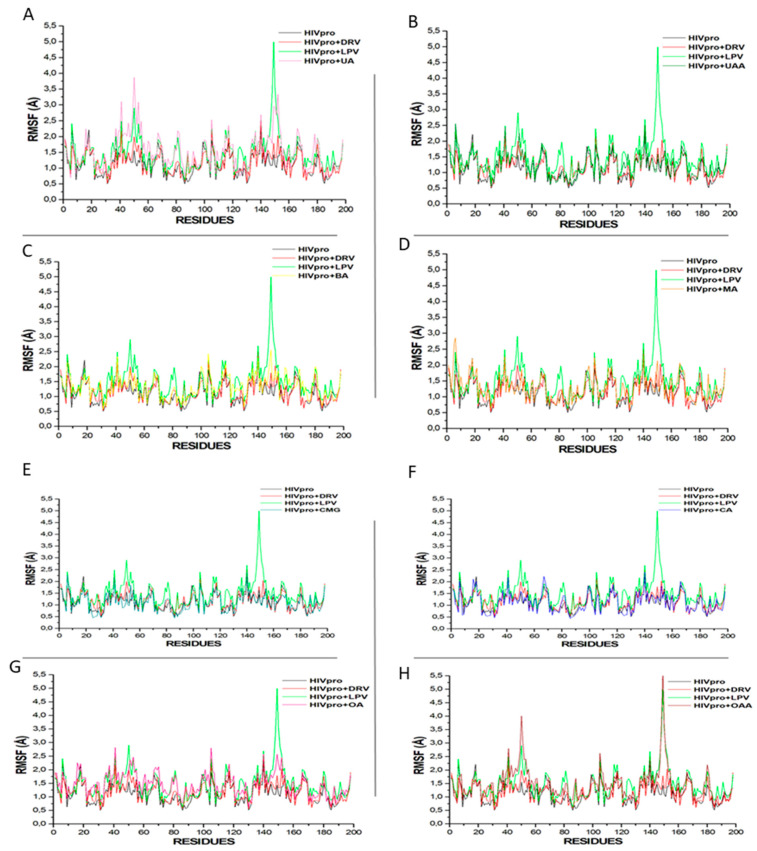
Comparative RMSF plots of residue-based average C-α fluctuations of free HIVpro and that bound with LPV, DRV, and (**A**) UA, (**B**) UAA, (**C**) BA, (**D**) MA, (**E**) CMG, (**F**) CA, (**G**) OA, and (**H**) OAA systems.

The RMSF measures the extent of the conformational flexibility of the ligand–receptor system following an MDS evaluation [39]. In this study, compared with other ligands, LPV, UA, and OAA exhibited greater protein flexibility at residues 40–60 and 140–160 (Figure 4). Similar increases in protein flexibilities were observed for the other compounds, with the HIVpro apo system having the lowest flexibility (Figure 4). However, it could be logically inferred that ligand binding increases the protein flexibility, with fluctuations at residues 45–55 and 145–155 (Figure 4), and these could be identified as the mirror residues in dimeric form of the HIVpro and may be substantive of the dimeric activity of the enzyme [34,35]. The fluctuation at residues 45–55 and 145–155 may also be suggestive of the opening and closing of the protease for ligand binding and interactions, as reported earlier by Kehinde et al. [34].

### 3.2. Solvent Accessible Surface Area (SASA)

**Figure 5:**
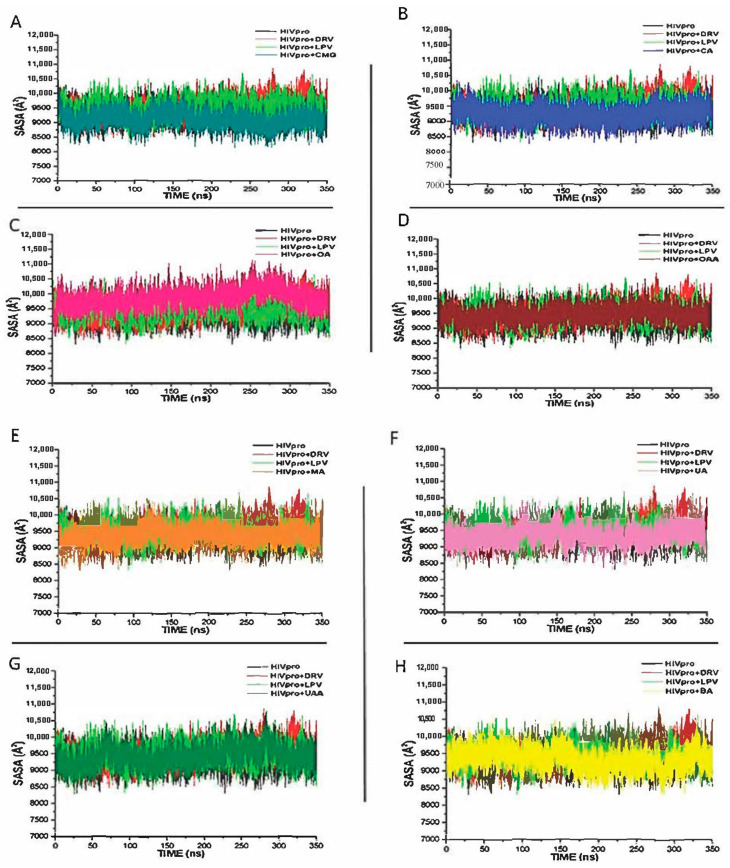
Comparative SASA plots of residue-based average C-α fluctuations of free HIVpro and that bound with LPV, DRV, and (**A**) CMG, (**B**) CA, (**C**) OA, (**D**) OAA, (**E**) MA, (**F**) UA, (**G**) UAA and (**H**) BAA systems.

The SASA is a key parameter in examining the impact of ligand binding on a receptor, evaluated through the receptor’s exposure to solvent molecules [40]. In this study, the binding of all the study compounds did not significantly change the SASA values of the bound systems in each case, relative to the free HIVpro, with the values obtained ranging from 8500 to 10,000 Å (Figure 5). This observation suggests that the structural integrity of the HIVpro was never compromised throughout the simulation period and that all the study metabolites conveniently bind and fit at the binding site of the enzyme. This may also justify why the binding energies of the metabolites fell in between those of the FDA-approved drugs.

### 3.3. HIVpro–Ligand Interaction

**Figure 6:**
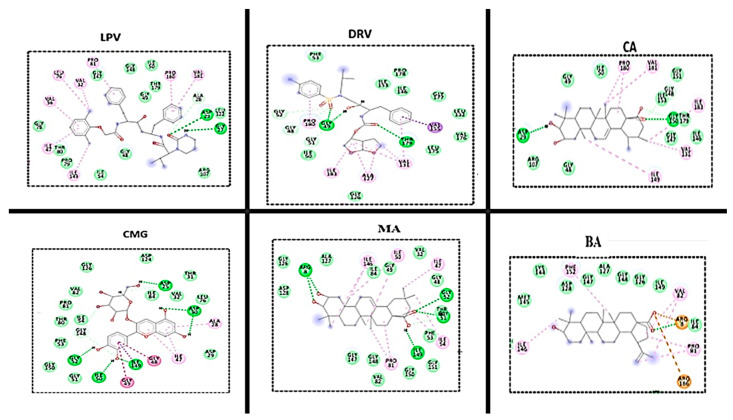
2D interaction plots of two reference drugs (DRV and LPV) and plant metabolites (BA, MA, CA, and CMG) with the active site amino acid residues of HIVpro.

Figure 6 shows the ligand interaction plots for the best four study compounds and the FDA-approved-drug-bound systems following the 350 ns trajectory. The types and number of interactions between proteins and ligands determine the overall binding free energy. Protein–ligand interaction has been widely used to examine the molecular interactions between residues at the active sites of protein and bound ligands [30,31]. The binding effect of different ligands on HIVpro was analysed, as well as the interaction between the key residues in the binding site in the presence of the two known inhibitors (DRV and LPV) and the selected metabolites. The results show that CMG and MA had a similar type of interaction with that of the FDA-approved drugs. This correlated with the high binding energy recorded for the two compounds, indicating that the two compounds are promising candidates for inhibiting HIVpro.

## 4. Conclusions

In this study, we investigated seven selected nutraceutical pentacyclic triterpenoids and an anthocyanin as potential inhibitors of HIV-1 subtype C protease enzyme (HIVpro) using MDS. The MMGBSA free energy calculations showed that the ΔG_bind_ of six lead ligands (CMG, MA, CA, BA, OA, and UAA) fell within the range of the two ΔG_bind_ of the reference FDA-approved drugs used in this study, with CMG and MA having a higher ΔG_bind_ than the conventional HIVpro inhibitors. Furthermore, the ligand interaction plots revealed that the metabolites interacted hydrophobically with the active site amino acid residues, with the identification of other key residues implicated in HIVpro inhibition for novel drug design. This is the first computational report on the anti-HIV-1 activities of CMG and MA, with efforts on their in vitro and in vivo evaluations underway. The study significantly revealed that the lead metabolites (CMG, MA, CA, BA, OA, and UAA) could be promising therapeutic agents against HIV-1. In addition, they could either be used alone or concomitantly with conventional HIVpro inhibitors such as LPV, DRV, cabotegravir (CBV), and rilpivarine (RPV) to improve their therapeutic activities against the HIV-1 serotype.

## Figures and Tables

**Table 1:** Docking scores for the two FDA-approved HIV-1 protease inhibitors and selected bioactive phytochemical compounds.

Compound Name	Docking Score (kcal/mol)
FDA-Approved Drugs	
LPV	−8.4
DRV	−8.1
Selected Bioactive Phytochemicals	
CMG	−9.9
MA	−9.3
CA	−9.0
BA	−8.3
OA	−8.6
OAA	−8.2
UAA	−8.5
UA	−8.1

**Table 2:** Thermodynamic energy components (kcal/mol) for the bioactive compounds and FDA-approved drugs to HIVpro after 350 ns MDS.

Complex	ΔE_vdw_	ΔE_elec_	ΔG_gas_	ΔG_solv_	ΔG_bind_
FDA-Approved Drugs					
LPV	−51.973 ± 5.433	−27.534 ± 6.605	−79.507 ± 7.958	−38.291 ± 3.540	−44.571 ± 3.952
DRV	−45.805 ± 6.108	−28.424 ± 8.120	−69.223 ± 10.871	−29.235 ± 4.206	−40.311 ± 4.943
Selected Bioactive Phytochemicals					
CMG	−37.080 ± 5.298	−41.112 ± 7.929	−78.176 ± 9.411	−20.285 ± 4.879	−57.890 ± 6.693
MA	−47.442 ± 4.300	−28.057 ± 6.689	−73.166 ± 9.794	−25.032 ± 4.845	−48.134 ± 6.002
CA	−45.738 ± 2.979	−19.633 ± 6.132	−67.884 ± 5.446	−23.306 ± 3.976	−43.900 ± 4.101
BA	−45.850 ± 4.123	−44.778 ± 9.576	−71.628 ± 8.503	−10.954 ± 2.467	−43.740 ± 4.288
OA	−39.596 ± 4.375	−5.091 ± 091	−56.095 ± 2.779	−8.940 ± 2.453	−42.010 ± 4.699
OAA	−39.4454 ± 6.256	−6.039 ± 1.909	−49.311 ± 7.672	−9.912 ± 4.453	−37.393 ± 6.001
UAA	−44.589 ± 4.054	−4.679 ± 10.634	−49.543 ± 4.265	−9.174 ± 3.586	−40.654 ± 2.705
UA	−45.761 ± 3.787	−7.069 ± 2.355	−51.754 ± 6.212	−11.589 ± 3.854	−40.165 ± 4.554

## Data Availability

Not applicable.
